# Getting to the heart of intraflagellar transport using *Trypanosoma* and *Chlamydomonas* models: the strength is in their differences

**DOI:** 10.1186/2046-2530-2-16

**Published:** 2013-11-29

**Authors:** Benjamin Morga, Philippe Bastin

**Affiliations:** 1 Trypanosome Cell Biology Unit, Institut Pasteur and CNRS, URA 2581, 25 rue du Docteur Roux, 75015, Paris, France

## Abstract

Cilia and flagella perform diverse roles in motility and sensory perception, and defects in their construction or their function are responsible for human genetic diseases termed ciliopathies. Cilia and flagella construction relies on intraflagellar transport (IFT), the bi-directional movement of ‘trains’ composed of protein complexes found between axoneme microtubules and the flagellum membrane. Although extensive information about IFT components and their mode of action were discovered in the green algae *Chlamydomonas reinhardtii*, other model organisms have revealed further insights about IFT. This is the case of *Trypanosoma brucei*, a flagellated protist responsible for sleeping sickness that is turning out to be an emerging model for studying IFT. In this article, we review different aspects of IFT, based on studies of *Chlamydomonas* and *Trypanosoma*. Data available from both models are examined to ask challenging questions about IFT such as the initiation of flagellum construction, the setting-up of IFT and the mode of formation of IFT trains, and their remodeling at the tip as well as their recycling at the base. Another outstanding question is the individual role played by the multiple IFT proteins. The use of different models, bringing their specific biological and experimental advantages, will be invaluable in order to obtain a global understanding of IFT.

## Review

Cilia and flagella are present at the surface of a large number of eukaryotic cells. They occur in two major kinds: motile and non-motile. They share a similar cylindrical structure of nine outer doublet microtubules, xtermed the axoneme. Most motile cilia exhibit the 9 + 2 structure in which the peripheral doublets surround a central pair of singlet microtubules. They possess molecular motors belonging to the dynein family that are responsible for ciliary movement. Immotile cilia usually have a 9 + 0 axonemal structure, which lacks the central pair of microtubules, the dynein motors, and other components involved in beating regulation such as radial spokes or the dynein regulatory complex. This structural organization is remarkably conserved throughout evolution from protists to mammals and is accompanied by molecular conservation [1,2]. Cilia and flagella are present in multiple protists, in many plant gametes (bryophytes, ferns, or gingko for example), and in animal tissues [3]. However, their number, length, and position can vary from one organism to another and even from one cell type to another in the same organism. For example in mammals, motile cilia are found on epithelial cells of the respiratory epithelium or the oviduct, whereas a single motile flagellum is encountered in spermatozoa. Specialized sensory cilia are present in neurons of the retina or of the olfactory epithelium [4,5]. In addition, the majority of mammalian cells have the ability to assemble a primary cilium that can be involved in chemical or mechanical perception [6].

Defects in assembly or functioning of cilia and flagella in humans lead to a wide variety of diseases called ciliopathies. Dysfunction of motile cilia was demonstrated more than 30 years ago and results in primary ciliary dyskinesia [7]. In the early 2000s, the malfunction of cilia was shown to be responsible for polycystic kidney disease in mice [8,9], revealing for the first time that primary cilia were not vestigial organelles as long thought but were actively involved in cell function [6,10]. Mutations in genes encoding ciliary proteins are now linked to a set of rare genetic diseases such as Bardet–Biedl syndrome, Alström syndrome, Jeune syndrome, asphyxiating thoracic dystrophy, and Meckel–Gruber syndrome to cite but a few [11].

Cilia and flagella are therefore highly important cellular structures and their absence is lethal for mammals [12], rendering studies in mouse models rather difficult, especially when dealing with organelle construction. Hence, scientists have used various model organisms to investigate the mechanisms of assembly of cilia and flagella [13]. In this review, we compare two models: the green algae *Chlamydomonas* and the protist *Trypanosoma*, in an effort to gain a more global view of the mechanisms governing flagellum construction.

### Chlamydomonas and Trypanosoma: two fascinating flagellated organisms

**Figure 1:**
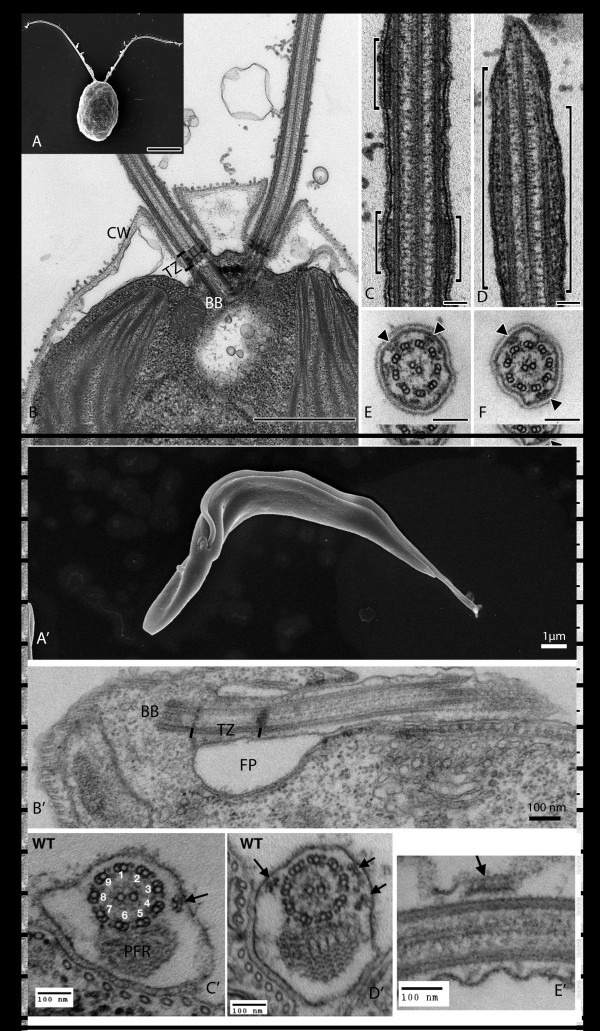
**Structure of the flagellum in *Chlamydomonas reinhardtii *and *Trypanosoma brucei.*** Scanning electron microscopy shows the global structure of **(A)***Chlamydomonas* and **(A’)***Trypanosoma*. Sections through the base reveal **(B)** the emergence of the two flagella from the cell wall (CW) and **(B’)** the single flagellum through the flagellar pocket (FP). The basal body (BB) and the transition zone (TZ) are also visible. **(C,D,E,F,C’,D’,E’)** Longitudinal and cross-sections through the flagellum reveal the structure of the axoneme (and of the PFR in *T. brucei*), and the presence of IFT trains are indicated by brackets or arrows. Scale bars: **(A)** 5 μm; **(B)** 1 μm; and **(C,D,E,F)** 0.1 μm. Image credit: **(A,B,C,D,E,F)** provided by Elisa Vannuccini and Pietro Lupetti (University of Siena, Italy). **(A’,B’)** reproduced with permission from Buisson and Bastin [15] and **(C’,D’,E’)** reproduced with permission from Absalon *et al*. [16]. The scale bar size is indicated on each image. BB, basal body; CW, cell wall; FP, flagellar pocket; IFT, intraflagellar transport; PFR, paraflagellar rod; TZ, transition zone.

*Chlamydomonas* is a unicellular photosynthetic eukaryote with an ovoid cell body that possesses two flagella (approximately 12 μm in length each) found at the apical end of the cell (Figure 1A,B). *Chlamydomonas reinhardtii* is a well-established model organism for studying fundamental processes such as photosynthesis, motility, responses to light, and cell-cell recognition. *Chlamydomonas* displays numerous biological and technical advantages for the study of eukaryotic flagella. First, it can be grown synchronously and large amounts of flagella can be easily purified for biochemical analyses. Second, forward genetics allows the generation of many mutant strains [14] that can be easily crossed for exhaustive characterization. Furthermore, *Chlamydomonas* cells exhibit complex swimming behaviors in response to various light stimuli, allowing dissection of flagellar beating regulatory pathways. Finally, flagella are not essential for the survival of *Chlamydomonas* but play key roles in gamete recognition, hence permitting investigation of sensory processes.

*Trypanosoma brucei* is a kinetoplastid protist that possesses one flagellum (approximately 22 μm in length) attached along the length of the cell body (Figure 1A’,B’). It is well known for being responsible for sleeping sickness in Africa. It proliferates in mammalian blood and is transmitted via the bite of a tsetse fly where it undergoes a complex development in the midgut and the salivary glands [17]. The flagellum remains present during the whole cell cycle and throughout the life cycle. The most extensively studied stage of this parasite is derived from the peritrophic space of the insect vector midgut, and is called the procyclic stage. The cell is 20 to 25 μm long and 3 to 5 μm wide and exhibits a slightly helical shape. *Trypanosoma* are attractive models to study cilia and flagella since they can be easily cultivated in laboratories and are genetically tractable (RNAi, endogenous tagging, imaging, and so on).

The flagellum of *Trypanosoma* and *Chlamydomonas* is of the 9 + 2 configuration (Figure 1E,F,C’,D’), as confirmed by the conservation of components of the dynein arms, the central pair, or the radial spokes [18,19]. In *Chlamydomonas,* the flagella emerge at the surface through pores in the cell wall (Figure 1B), whereas in trypanosomes, the flagellum arises from a cell surface invagination called the flagellar pocket (Figure 1B’) that is also the unique site for endocytosis and exocytosis [20]. Nevertheless, there are some significant differences such as the presence of an extra-axonemal structure called the paraflagellar rod (PFR) in the *Trypanosoma* flagellum (Figure 1C’,D’). This unique structure is made of filaments finely organized and can be subdivided into three distinct regions called the proximal, intermediate, and distal domains [21]. The PFR is composed of unique proteins and is required for cell motility, although its actual contribution to flagellum beating remains enigmatic [22-24]. Some ultrastructural differences have been noted at the base of the flagellum: the transition zone is longer in trypanosomes (approximately 400 nm) (Figure 1B’) and does not contain the central structure encountered in *Chlamydomonas* (Figure 1B). Some differences have also been reported at the distal tip where the central pair of microtubules is linked to the membrane by a cap in *Chlamydomonas*[25], whereas a discrete electron-dense structure more distant from the membrane is found in *Trypanosoma*[26].

**Figure 2:**
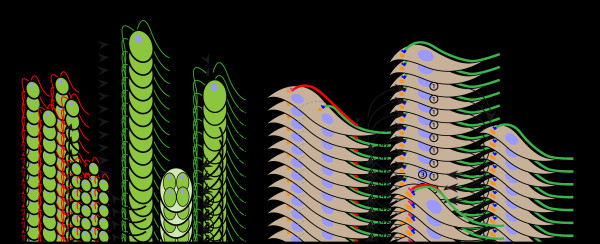
**Mode of flagellum formation and relationship with the cell cycle. (A)** In *Chlamydomonas*, the flagella are disassembled prior to mitosis during the asexual life cycle. **(B)** In the procyclic form of *Trypanosoma brucei*, a new flagellum is constructed while the old one remains in place. Mature and assembling flagella are shown in green and red, respectively (see text for details). The tip of the elongating flagellum is indicated with arrowheads and the plane of cleavage is shown by dotted lines.

The coordination between cell division and flagellum formation is rather divergent between *Chlamydomonas* and *Trypanosoma* (Figure 2). In *Chlamydomonas*, a vegetative cell divides within a single cell wall to produce 4, 8, 16, or more cells from a unique parent. During mitosis flagella are resorbed and basal bodies ensure their function as centrioles, organizing the mitotic spindle [27]. Mitosis is closed, meaning that the nuclear membrane does not break down. Instead, microtubules cross the nuclear envelope through its pores to assemble the mitotic spindle. The new flagella are assembled once the cell has fully divided (Figure 2A). In the trypanosome cell division cycle, two distinct S phases need to be coordinated: one for the mitochondrial DNA contained within the kinetoplast (trypanosomes possess a single mitochondrion) and one for nuclear DNA (Figure 2B). The process begins with the S phase of the mitochondrial DNA immediately followed by basal body maturation and duplication [28,29]. A tripartite filament system connecting the duplicated DNA and a specific membrane region of the mitochondrion is duplicated and linked to the duplicated basal bodies [30]. The old flagellum remains in place and the new flagellum invades the flagellar pocket and connects its tip to the old flagellum by a transmembrane mobile junction called the flagella connector (FC) [31,32]. It has been proposed that the FC guides the positioning of the new flagellum. Then, mitosis occurs leaving one of the two nuclei positioned between the two kinetoplasts, to finally produce two ‘clusters’ of cytoplasmic organelles ready for division. Cleavage furrow ingression is unidirectional, from the anterior to the posterior end of the dividing cell, between the old and the new flagellum. The length of the new flagellum determines the point where cell cleavage initiates and hence the length of the daughter cell (Figure 2B) [33].

### Construction of the flagellum: a 500-piece jigsaw

The assembly of the flagellum is a huge commitment for the cell, as this requires the correct production and assembly of more than 500 proteins [18,19,34], both in time (right moment of the cell cycle) and in space (in a defined compartment). Assembly of the axoneme [35,36] and also of the PFR [37] takes place at the distal end of the growing flagellum. Since the flagellum does not possess any ribosomes, all the components needed for its construction must first be synthesized in the cytoplasm and then imported into the flagellum before reaching the distal tip either by transport or by diffusion. In 1993, an active transport of ‘rafts’ was discovered within the flagellum of *Chlamydomonas* and termed intraflagellar transport (IFT) [38]. It was first observed by differential interference contrast (DIC) microscopy in paralyzed flagella of live cells. It was proposed that these rafts could correspond to electron-dense structures sandwiched between the flagellar membrane and the axonemal outer doublet B identified by electron microscopy in the late 1960s [39]. IFT was not observed in the thermosensitive Fla10 mutant [40] maintained at the restrictive temperature and the number of particles detected by electron microscopy dropped significantly, supporting the proposal that these electron-dense structures indeed correspond to the transported granules detected by DIC [41]. These were termed IFT particles and later renamed as IFT trains [42]. Fla10 is a kinesin motor member of the heterotrimeric kinesin-2 complex composed of two motor subunits (FLA10 and FLA8) and a kinesin-associated protein (KAP) possibly involved in cargo binding [43]. Immunogold experiments revealed that FLA10 localizes to the particles [41].

This discovery raised the question of the identity of the molecules involved in this transport. Cole and co-workers were the first to purify the IFT particles from the flagellum matrix of *Chlamydomonas* using sucrose density gradients [44,45]. Two distinct complexes (A and B) were identified: the IFT-A complex is a 550-kDa tetramer containing at least five subunits of 144, 140, 139, 122, and 43 kDa, whereas IFT-B complex is a 750-kDa complex containing at least 11 subunits ranging from 20 to 172 kDa (Table 1) [45]. A metagenomic analysis revealed that most *IFT* genes are conserved in ciliated and flagellated species [1,46] with the exception of *Plasmodium* that assembles its flagella in the cytoplasm [47]. In most species, IFT proteins are found in the flagellum but are mostly concentrated at its base and also found in fairly high abundance in the cytoplasm [48,49]. Mutant analysis demonstrated that kinesin-2 is responsible for anterograde movement [41], whereas retrograde trafficking is powered by a specific type of dynein motor [50-52]. *IFT* genes are conserved in all trypanosomatid genomes (Table 1) with the exception of the *KAP* that is missing [53], suggesting that kinesin-2 is more likely to function as a homodimer as reported for the osm-3 kinesin in *Caenorhabditis elegans*, and not as a heterotrimer as observed in other species [54]. In contrast, the *Chlamydomonas* genome appears not to contain *osm-3* homologues, indicating that only heterotrimeric kinesin-2 is present in this organism.

**Table 1:** Role of various IFT proteins and motors

**Protein name**	***Chlamydomonas***	***Trypanosoma***
**Kinesin II**		
KIN2A	A *(fla10)*	A
KIN2B	A *(fla8)*	A
KAP	A *(fla3)*	X
**IFT dynein**		
DHC1b/DHC2.2	R (*)	R
DHC2.1	X	R
D1bLIC	R (*)	R
FAP133	-	R
LC8	-	-
**IFT-A**		
IFT144	R *(fla15)*	R
IFT140	-	R
IFT139	R *(fla17)*	-
IFT122	-	-
IFT121	-	R
IFT43	-	-
**IFT-B**		
IFT172	A/R *(fla11)*	A
IFT88	A (*)	A
IFT81	-	A
IFT80	-	-
IFT74	-	-
IFT70/PIFTB2	A	A
IFT57	-	A
IFT54	-	A
IFT52	A *(bld1)*	A
IFT46	A (*)	-
IFT27	?	R
IFT22/RABL5	?	R
IFT20	-	A
PIFTA1/FAP22/DYF3	-	A
PIFTC3/DYF13	-	A

Proteins have been grouped according to their category. When inhibition of a given gene blocked flagellum formation, it was described as an anterograde phenotype (A) and when it stopped retrograde transport, resulting in the formation of shorter flagella filled with IFT material, it was described as a retrograde phenotype (R). Ambiguous phenotypes are shown with a question mark and a hyphen indicates that the gene is conserved but has not been studied in that organism. A cross indicates that the gene is missing from the genome. Experiments were carried out by RNAi knockdown except where indicated. The asterisk denotes an insertion mutant whereas names of the mutant gene obtained by forward genetics are shown in italics

IFT plays a key role in the construction of the flagellum as its inactivation blocks flagellum formation in all species studied so far. Inactivation of any single *IFT* gene is sufficient to inhibit flagellum assembly, indicating that the integrity of the particle is required for efficient IFT. This is supported by many experiments using mutant, RNAi, or knockout approaches in different organisms: *Chlamydomonas*[41], mouse [55], *C. elegans*[56], *Tetrahymena*[57], *Trypanosoma*[33], zebrafish [58], *Leishmania*[59], and *Xenopus*[60].

**Figure 3:**
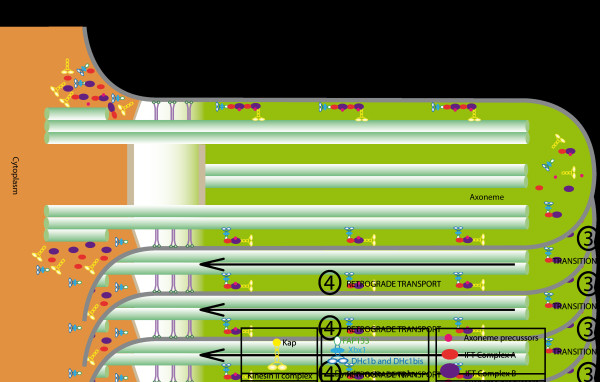
**Canonical model for IFT.** Step 1: IFT-A and IFT-B complexes, kinesin-2, and inactive cDynein1b gather at the base of the flagellum. Step 2: the active kinesin-2 transport IFT-A and IFT-B complexes, inactive cytoplasmic dynein 2, and axonemal precursors from the base to the tip. Step 3: kinesin-2 reaches the distal end, where axonemal cargo proteins and IFT particles are released into the ciliary tip compartment. IFT-A and IFT-B complexes dissociate from each other. Complex A binds to active cytoplasmic dynein 2. Step 4: active cytoplasmic dynein 2 transports complexes IFT-A and IFT-B and kinesin to the cell body. IFT, intraflagellar transport.

The currently accepted model for IFT mostly relies on studies of *Chlamydomonas*[61] and is summarized in Figure 3. First, IFT-A and IFT-B complexes, kinesin-2, cytoplasmic dynein 2, and axonemal precursors are produced in the cytoplasm and gather at the flagellum base. Second, once inside the flagellum, the active kinesin-2 transports IFT-A and IFT-B complexes, inactive IFT dynein, and axonemal precursors from the base of the flagellum to the tip. Third, kinesin-2 reaches the distal end of the B microtubule, where axonemal cargo proteins and IFT particles are released into the ciliary tip compartment. Following remodeling of the IFT train, complex A binds to the active IFT dynein. Fourth, the IFT-B complex associates with the IFT-A complex, and the active IFT dynein transports all components, including kinesin-2 back from the tip to the cell body. The IFT cycle is completed when IFT components are returned at the base of the flagellum, where they can be recycled or targeted for destruction.

### Setting-up the scene

**Figure 4:**
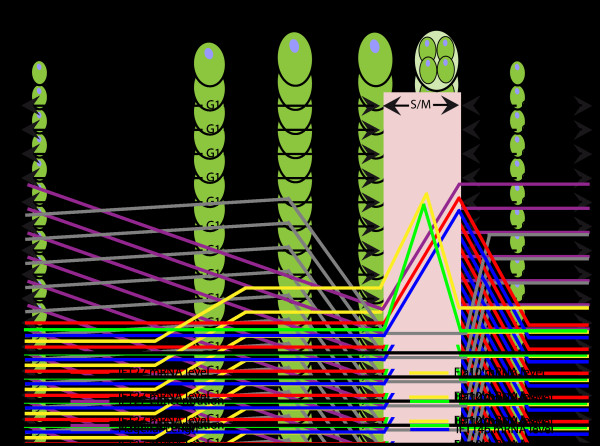
**Evolution of the amounts of various *IFT *mRNA during the cell cycle in *Chlamydomonas*.** The relative concentrations of IFT27 protein (purple line) and *IFT27* mRNA (red line), *IFT46* mRNA (blue line), *IFT140* mRNA (green line), and *Fla10* mRNA (yellow line) are plotted along with flagellum length (solid grey line). IFT27 protein concentration decreases continuously during G1 and reaches its lowest level just before division. *IFT27*, *IFT46*, *IFT140*, and *Fla10* mRNA and protein are normally synthesized during S/M which resets its levels for the next cell cycle. Figure modified from Wood *et al*. [64]. IFT, intraflagellar transport.

Producing at least 500 proteins at about the same time and same place is a sophisticated feat of engineering. This includes proteins constituting the basal body, the transition zone, the IFT particles, and the axoneme (and the PFR in trypanosomatids), as well as membrane elements. In *Chlamydomonas*, deflagellation stimulates the transcription of all flagellar genes [62]. This is accompanied by a stimulation of synthesis of flagellar proteins through an increase in the level of translatable mRNAs. The stimulation of the production of mRNA could be related to the presence of response elements called ‘tub boxes’ found in the promoter region of several flagellar genes [63]. Monitoring of some IFT proteins during the normal cell cycle in *Chlamydomonas* has brought more insight about the timing of this process [64]. After cell synchronization, it was found out that transcripts for IFT27, IFT46, IFT140, and FLA10 were upregulated during the S/M phase before the construction of the flagellum (Figure 4). Another study has shown that mRNAs for tubulin and other axonemal components such as radial spoke and outer or inner dynein arms were overexpressed during flagellar regeneration [65].

**Figure 5:**
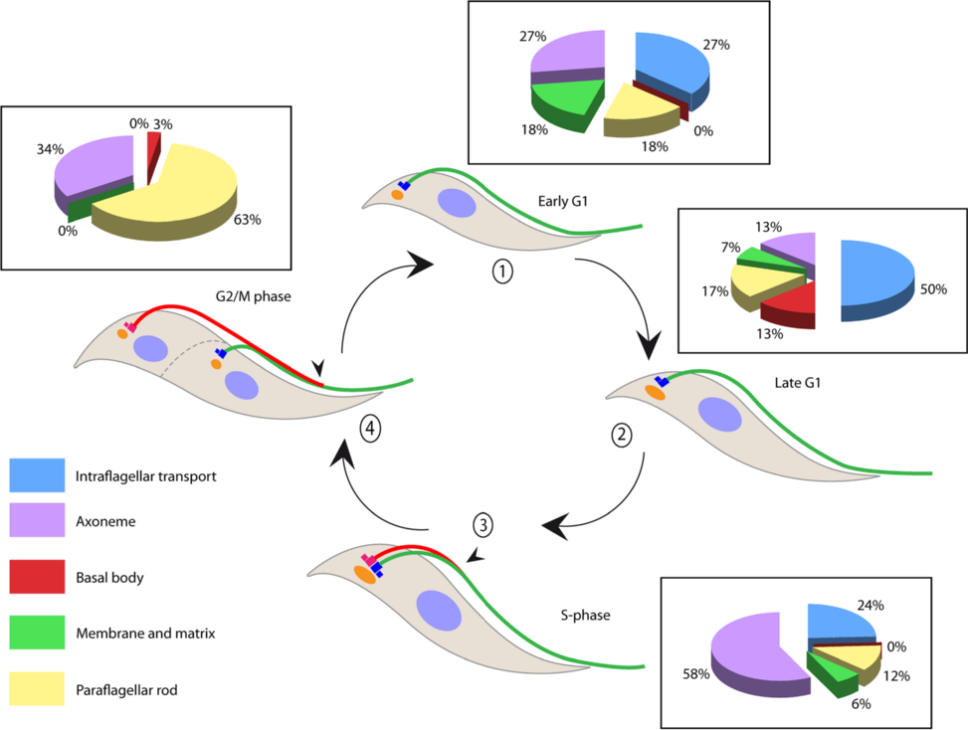
**Expression of the mRNA encoding flagellar proteins during the *Trypanosoma brucei *cell cycle. (1)** Early G1: cells with one flagellum. **(2)** Late G1: maturation and duplication of the basal body. **(3)** S phase: construction of the new flagellum. **(4)** G2/M phase: elongation of the new flagellum. IFT, basal body, and membrane and matrix genes peak first, whereas axoneme and PFR transcripts emerge later when flagellum elongation takes place. Original data are from Archer *et al.*[66] and transcripts encoding proteins belonging to different structures are listed in Additional file 1: Table S1. IFT, intraflagellar transport; PFR, paraflagellar rod.

Until recently, little was known about the expression of mRNA encoding flagellar proteins in *T. brucei* due to difficulties of reliably synchronizing cells in culture. However, a recent study overcame this limit, allowing the investigation of the gene expression profile during the cell cycle [66]. Procyclic *T. brucei* cells were collected in log phase and treated by elutriation in order to separate cells by density and size. The larger cells were collected and placed for 1 hour in culture, and then a second elutriation centrifugation was performed in order to select the smallest cells that had just divided. These were returned in culture and proceeded with good synchronization through the cell cycle that was completed in 9 hours [66]. An RNA-seq profile using Solexa sequencing (Illumina, San Diego, CA, USA) was performed at four stages: early G1 (cells with one flagellum), late G1 (maturation and duplication of the basal body), S phase (construction of the new flagellum), and G2/M phase (elongation of the new flagellum). A total of 546 genes showed cell cycle-dependent fluctuations, with peaks at specific time points (Figure 5). Since many of them encode components of flagellum structures, we analyzed the list in detail and grouped genes according to their relation to basal body, IFT, membrane and matrix, axoneme, and PFR (Additional file 1: Table S1). Analysis of gene expression profiles revealed that most of the basal body mRNAs were upregulated when the basal body was duplicating (late G1). This phase was preceded by a peak in mRNA for IFT and membrane proteins prior to the initiation of flagellum construction. The mRNAs for axoneme components were mainly produced when the new flagellum elongates, whereas the *PFR* mRNA increased later on (Figure 5). This is consistent with the fact that this structure is the last one to be assembled in the flagellum [67]. Therefore, the profile of mRNA production correlates with the successive steps of flagellum formation, suggesting that trypanosomes produce the right amount of transcripts exactly when needed. Protein translation is expected to follow the RNA dynamics of production although direct evidence is missing. These observations are in agreement with the fact that the actual amount of flagellar proteins available in the cytoplasm is very low [23]. This situation is quite different from *Chlamydomonas* where a pool of non-assembled material is available in the cytoplasm and sufficient to support construction of two half-length (or one full-length) flagella [68].

### Putting pieces together

Construction of the flagellum follows a strict hierarchy: maturation of the basal body, docking to the membrane, formation of the transition zone, and then elongation of the axoneme. In trypanosomes, the first detectable event in the cell cycle is the maturation of the probasal body that elongates and docks to the membrane via the transition fibers, becoming competent for nucleating the new flagellum. This maturation process is concomitant with the formation of a new probasal body alongside each mature basal body [28]. Such a cell possesses two basal body complexes assembled at the same time, but the mature basal body, which bears the old flagellum, is always at least one generation older than the one that bears the new flagellum. In *Chlamydomonas*, the existing flagella are disassembled at mitosis, but old and new basal bodies differ slightly in their protein composition. The docking of the basal body is independent of IFT since it takes place apparently normally in all IFT mutants [16].

**Figure 6:**
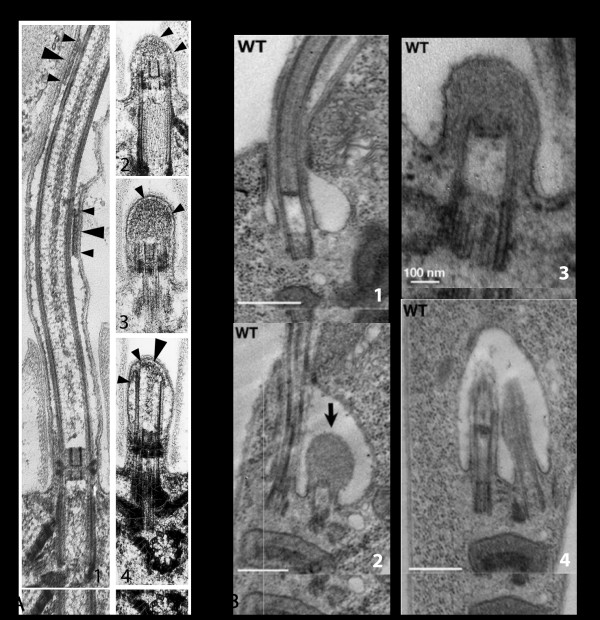
**An accumulation of electron-dense material precedes flagellum elongation. (A)** Cross-sections through the flagellum base of *Chlamydomonas* cells that undergo regeneration fixed shortly after pH shock-induced deflagellation. IFT particles (arrowheads) are visible in all flagella. In short flagella, numerous particles fill the space distal to the basal body, but by the time microtubules have formed (D), particles have become organized to form linear arrays. IFT particles are linked to the microtubules (small arrowheads) and to the membrane (small arrowheads). Scale bars: 0.1 μm. Reproduced with permission from Dentler [74]. **(B)** Cross-sections through the flagellar pocket in which the new flagellum is built in procyclic *Trypanosoma brucei.* The short new flagellum contains a large amount of electron-dense material, while microtubules are not yet assembled. Once microtubules have started to elongate, this material is much more discrete. Scale bars: 500 nm, except where indicated. Reproduced with permission from Pazour *et al*. [18]. IFT, intraflagellar transport.

At the early stage of flagellum formation, a large amount of electron-dense material is observed by transmission electron microscopy (TEM) in the short flagellum of both *Chlamydomonas* and *Trypanosoma*, prior to microtubule elongation (Figure 6). The identity of this material remains to be determined. It could correspond to tubulin and other axoneme precursors before their assembly or to IFT material. This hypothesis is supported by immunofluorescence assays in *Trypanosoma* showing a bright signal for IFT proteins in short flagella before axoneme markers can be detected (T Blisnick, unpublished data). Similarly, immunofluorescence assay (IFA) with an anti-IFT52 antibody and live microscopy analysis of GFP::IFT27 expressing cells data show that a high concentration of IFT protein is present at early stages of flagellum formation in *Chlamydomonas*[36,69]. Immunoelectron microscopy indicates that IFT52 is associated with the periphery of transitional fibers, which extend from the distal portion of the basal body to the cell membrane and demarcate the entry of the flagellar compartment [70].

There is very little information about the way IFT trains are assembled within the flagellum. The IFT-A and IFT-B complexes can be purified from cell bodies in *Chlamydomonas* suggesting that they are preassembled in the cytoplasm [71]. In *Trypanosoma*, as in *Chlamydomonas*, IFA or biochemical fractionation indicate that a large amount of IFT protein is present in the cytoplasm [48,49]. Quantification experiments revealed that the cell body contains up to 50-fold more IFT material than the flagellum [48]. Nevertheless, conventional IFT trains have never been visualized elsewhere other than in the flagellum compartment. In *Trypanosoma*, GFP::IFT52 is found at the flagellum base and traffics in the flagellum, but is also very abundant in the cytoplasm. Photobleaching an area of the cytoplasm resulted in rapid recovery but no train movement could be detected (J Buisson, unpublished data).

We propose that IFT train formation takes place when the local concentration of IFT complexes is sufficiently high. This might only be achieved at the very early phase of flagellum formation, when IFT proteins appear to be highly concentrated in the short flagellum (Figure 6). Dynein is not required for this process since long trains can be incorporated in the short flagellum of the *fla14* mutant, that present a mutation in a dynein light chain [42]. In the future, it will be interesting to produce *in vitro* the IFT complexes [72,73] and the two different motor complexes, to monitor their ability to constitute trains according to their respective concentration and the nature of the environment.

### System in action

*In vivo* visualization of IFT particles is essential to understand the mechanisms responsible for flagellum growth and maintenance. This can be achieved by two methods: direct observations by DIC (so far only achieved in *Chlamydomonas*) and by the use of IFT proteins fused to fluorescent markers such as GFP. When viewed in DIC, IFT trains in *Chlamydomonas* leave traces on kymographs that appear twice as large (0.12 μm) for anterograde trains compared to retrograde ones (0.06 μm) [74]. However, these should be viewed as approximations given the limited resolution of light microscopy. More recent electron tomographic analysis of IFT trains *in situ*[42] discriminated two populations. An electron-opaque population gathers around a 250 nm size and exhibits an approximate 16 nm periodicity, and a less electron-opaque type with a 700 nm mean length presents an approximate 40 nm periodicity. Longitudinal sections of *fla14* flagella only showed long trains of low electron density and/or presenting a 40 nm periodicity [42,51]. Therefore, long trains are likely to correspond to anterograde particles and short trains represent the retrograde IFT trains. Hence, DIC appears to underestimate the actual size of the IFT trains. In *Trypanosoma*, analysis of the traces left by GFP::IFT52 from live cells suggests that the trains are at least 400 nm in length on the anterograde transport direction and 250 nm in the retrograde direction. These must be considered as approximations due to the limited resolution of light microscopy and the relatively long exposure time [75]. In the original publication, Kozminski and co-workers reported IFT rates of 2.0 μm/s^**-1**^ in the anterograde direction and 3.5 μm/s^**-1**^ in the retrograde direction. However, some variability has been observed between different experiments (Table 2). In *Trypanosoma*, IFT is sensitive to temperature (Table 2). Hence, fluctuations in the reported IFT speed could be related to experimental conditions, particularly because it is difficult to regulate or measure temperatures when observing IFT with oil immersion lenses and high intensity illumination.

**Table 2:** **IFT parameters in *Chlamydomonas *** **and *Trypanosoma***

**Reference**	**Parameter**	**Anterograde μm/s**^**-1**^	**Retrograde μm/s**^**-1**^
***Chlamydomonas***			
Kozminski *et al.*[38]	DIC	2.0	3.5
Iomini *et al.*[76]	DIC	1.8	3.1
Dentler [74]	DIC	1.9	2.8
Qin *et al.*[77]	IFT27	1.9	3.3
Engel *et al.*[69]	KAP	2.3	nd
	IFT27	2.2	nd
	DIC	2.4	nd
Lechtreck *et al.*[78]	IFT20	2.1	2.5
***Trypanosoma***			
Buisson *et al.*[75]	IFT52 (27°C)	2.4 (1.5)*	5.6
Buisson *et al.*[75]	IFT52 (37°C)	3.2 (2.2)*	7.4
Buisson *et al.*[75]	DHC1b (27°C)	2.3 (1.3)*	5.0

Asterisks indicate the population of slower anterograde trains. DHC, dynein heavy chain; DIC, differential interference contrast; IFT, intraflagellar transport; KAP, kinesin-associated protein; nd, no data.

#### The outbound journey

Whereas heterotrimeric and homodimeric kinesin-2 complexes are present in metazoans, *Chlamydomonas* and *Trypanosoma* possess only the heterotrimeric and the homodimeric, respectively, making them ideal models to study each complex individually. Analysis of *fla10*, *fla8*, and *fla3* mutations revealed changes in the rate, frequency, and processivity of anterograde IFT, ultimately leading to its cessation [41,76,79,80]. Mutation in the *fla10* and *fla3* genes results in the production of a less stable protein that is sensitive to temperature, thereby allowing an easy comparison of normal and mutant situations. In *Trypanosoma,* two genes encode for kinesin-2 motor submits but no KAP orthologue could be found in the genome [53,81]. Their function is currently being deciphered, indicating involvement in flagellum biogenesis (authors’ unpublished data).

Kinesin-2 transports the IFT-A and IFT-B complexes as well as the dynein motor. The exact organization of these four complexes during train formation and transport remains to be established. Immunoprecipitation data from flagellar extracts in *Chlamydomonas* indicate interactions between the different elements [61]. Interfering with any complex B polypeptide in *Chlamydomonas*, as in *Trypanosoma*, almost invariably leads to inhibition of cilia assembly, leading to the notion that the IFT-B complex is required for anterograde transport (Table 1). This could take place by several means. First, the IFT-B could be a central component of the train and its loss would prevent formation of new trains. Second, it could be required to activate the kinesin-2 motor. Third, it could control the entry in the flagellum since large proteins do not diffuse freely [82]. In all three cases, interfering with the IFT-B complex would lead to the inhibition of IFT. An intriguing exception is IFT22/RABL5, a protein that purifies with the IFT-B complex in both *Chlamydomonas* and *Trypanosoma*[83,84], for which RNAi silencing in *Trypanosoma* results in the formation of short stumpy flagella filled with IFT particles, a phenotype typical of retrograde defects [85]. A related phenotype has been reported recently in *Chlamydomonas*[84]. This indicates that at least one of the IFT-B proteins could participate to other processes than anterograde transport.

#### The switch

Observations in live cells revealed that once trains arrive at the tip of the flagellum, they are rapidly recycled into retrograde trains. Little or no accumulation of IFT material has been reported at the tip. In *Trypanosoma*, Buisson and co-workers have shown by photobleaching experiments that IFT proteins spend on average 3 to 4 seconds at the tip. What happens to the trains during this transition to retrograde transport?

We can propose at least four hypotheses. First, once kinesin-2 reaches the end of the axoneme, the motor falls off the microtubule and the dynein is targeted to another microtubule bringing the unmodified train for the return trip. However, this proposition is not compatible with TEM analysis where anterograde trains appear three-fold longer than retrograde trains [42]. Second, the train could change configuration at the tip to become more compact, behaving like a spring or being bent in two or more layers. This would be coherent with the 3D surface rendering that look clearly different between the two types of trains [42] but is difficult to reconcile with trafficking data in live cells. Indeed, if trains contain the same amount of material on a shorter surface, the signal intensity for the GFP IFT fusion proteins should look brighter. Yet this is the opposite that is observed for all IFT proteins or motors studied so far in both *Chlamydomonas* and *Trypanosoma*[69,75,77]. Third, when trains arrive at the tip of the flagellum, they could be fragmented in smaller trains, a hypothesis supported by the 3:1 ratio of retrograde/anterograde events measured on videos of GFP::IFT52 in *Trypanosoma*[75]. This is in agreement with electron microscopy data of *Chlamydomonas* that show anterograde trains are almost three times longer than retrograde trains. However, these results are not compatible with the DIC observations. This could be explained if some trains escape detection because they are too small or badly positioned. The actual sizes of trains reported by DIC is 0.06/0.12 μm [74], thus much shorter than detected by TEM, a feature probably related to the resolution limit issue raised above. It would therefore be no surprise if smaller trains were not detected. Finally, all the anterograde trains might not be recycled and some of them could be destroyed (degraded) or secreted out. Intriguingly, secretion was reported at the tip of the flagellum of *Chlamydomonas*[86-88]. Recently, Dentler has shown the importance of the secretory pathway to assemble and maintain full-length flagella in *Chlamydomonas*[89].

#### The inbound journey

The motor that powers retrograde IFT is called cytoplasmic dynein 2 or IFT dynein. This motor complex consists of at least four different subunits: a heavy chain (DHC1B/DHC2) that belongs to the AAA + family of ATPases, a light intermediate chain (DYNC2LI1/LIC/XBX1), a light chain (LC8), and a recently identified putative intermediate chain (IC/FAP133) containing WD repeats [90-92]. The heavy chain was originally identified because its synthesis was induced by deciliation in sea urchin embryos [93]. Mutations in *Chlamydomonas* or RNAi knockdown in *Trypanosoma* of any of these genes result in a phenotype consistent with defective retrograde IFT [33,49-52,80]. One intriguing particularity of all trypanosomatid genomes is the presence of two different genes coding for the dynein heavy chain. The divergence between their sequences indicates that the duplication must be a rather ancient event. Amazingly, these two heavy chains are not redundant since any single gene is essential for retrograde transport (T Blisnick *et al*., unpublished data). The significance of this observation remains to be clarified.

In contrast to IFT-B proteins, IFT-A polypeptides are not always essential for building the ciliary axoneme, but rather, are important for retrograde IFT. Several *Chlamydomonas* mutants that contain decreased amounts of IFT-A polypeptides are still able to assemble flagella of almost normal length but display accumulation of IFT-B complex polypeptides at the tip [76,94,95]. More pronounced phenotypes were observed upon RNAi knockdown in *Trypanosoma*[16] where only a very short flagellum was constructed with a normal basal body and transition zone, but with a spectacular accumulation of electron-dense material dilating the flagellum. The axoneme was highly disorganized and even split. These differences could be explained by the nature of the *Chlamydomonas fla15* (IFT144) and the *fla17* mutations that correspond to a point mutation and to a short truncation, respectively, hence corresponding to a hypomorphic rather than a null mutation [94].

The way by which the IFT-A complex contributes to IFT remains elusive. Different possibilities could be considered. First, the IFT-A complex could associate to the IFT dynein either to ensure its transport during the anterograde trip or for proper functioning in the retrograde event. Second, the IFT-A complex could intervene at an earlier stage, for example by controlling the entry of IFT dynein in the flagellum. Third, it could participate to the inactivation of the kinesin motor during the switch phase. Finally, the IFT-A complex could control the remodeling of the train at the tip of the flagellum. In all cases, its inhibition would result in the accumulation of IFT-B complex proteins at the end of the flagellum. It becomes increasingly important to understand the actual function of the IFT-A complex since numerous mutations affect *IFT-A* genes in patients suffering from various ciliopathies. Remarkably, none of the mutations are expected to yield a null phenotype but are rather discrete modifications of the protein sequence [96-99].

#### How to deal with bidirectional transport?

IFT is a bidirectional movement of fairly large protein complexes in the narrow space between the microtubules and the flagellum membrane. The visualization of IFT in *Trypanosoma* and *Chlamydomonas* with fusion GFPs has shown the absence of visible collisions between anterograde and retrograde trains [69,75,76].

A simple explanation would be to consider that nine microtubule doublets are available for trafficking and that there is enough room for trains to cross, despite the high frequency of anterograde and retrograde events. An alternative hypothesis consists of using specific and distinct sets of microtubule for anterograde and retrograde trains, exactly as in a train system where outbound and inbound trains use their own tracks. Examination of cross-sections of the *T. brucei* flagellum revealed that IFT trains are restricted to two sets of specific doublet microtubules (3 to 4 and 7 to 8) (Figure 1C’,D’) [16]. This could be partially explained by physical constraints resulting from the presence of the PFR that could restrict the movement of IFT molecular motors and their cargo along microtubules. However, IFT particles are virtually never encountered close to doublets 1, 2, and 9 at the complete opposite of the PFR where access is not an issue [16]. Therefore, we propose that some doublets serve as specific tracks for anterograde or retrograde transport, hence reducing the risk of collision and offering the opportunity for precise and specific regulation of each set of motor. This has not been established in *Chlamydomonas* but it seems easy to do given the absence of outer dynein arm (ODA) on doublet 1 [100], providing a landmark for microtubule numbering. In the future, it will be exciting to establish the exact positioning of anterograde and retrograde IFT trains along axonemal microtubules.

#### Recycling is natural

**Figure 7:**
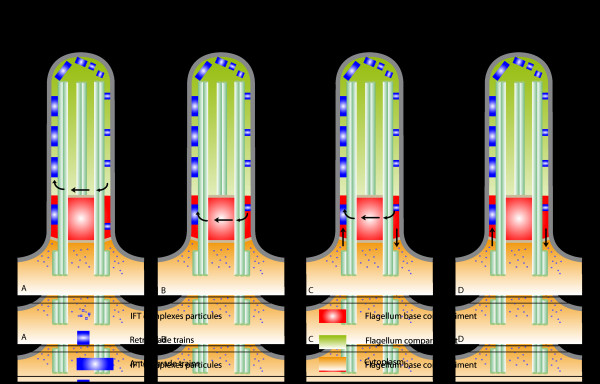
**Four different models illustrating the possible fate of IFT trains after they are returned to the base of the flagellum. (A,B)** Closed model, **(C)** semi-open model, and **(D)** open model (see text for details). Large blue boxes, anterograde trains; small blue boxes, retrograde trains; and blue dots, IFT complexes particles. The orange, red, and green colors indicate the cytoplasmic, flagellum base, and flagellum compartment, respectively. IFT, intraflagellar transport.

IFT trains travel to the tip of the flagellum and back to the base, but the fate of the IFT material once it is returned to the base has been little investigated. Taking into consideration the existence of three different pools of IFT material: in the flagellum, at the flagellum base, and in the cytoplasm, three different situations can be considered where the IFT system is closed, semi-open, or open (Figure 7). First, all IFT proteins are piled up in a flagellum at the beginning of its construction [36,101] and this material does not exchange with the cytoplasm. IFT trains could be either directly recycled to the flagellum compartment without exchange with the flagellum base material (Figure 7A) or they could return to the pool at the base of the flagellum and exchange (or queue) with IFT material concentrated in there before reiterating a cycle in the flagellum (Figure 7B). In the semi-open model, some trains could be mixed with the pool at the flagellum base pool and recruited to make new trains, whereas others could be discarded in the cytoplasm and replaced by fresh IFT proteins (Figure 7C). Finally, the open model implies that trains are used only once and exit the flagellum to be replaced by IFT proteins coming from the cytoplasm (Figure 7D).

Fluorescence recovery after photobleaching (FRAP) of the GFP::IFT52 fluorescent signal found at the base of the trypanosome flagellum was used to investigate IFT protein dynamics [75]. Photobleaching was followed by a period where no new fluorescent trains entered the flagellum, arguing against the first model. Fluorescent signal was recovered at the flagellum base with a kinetic dependent on the time fluorescent trains spent in the flagellum. Recovery was faster in shorter flagella or when the IFT rate was increased by shifting cells to 37°C [75]. Quantification data were in agreement with a ‘mix and queue’ model at the flagellum base, revealing that only half of the pool was directly involved in IFT. However, the results could also be explained by an equilibrated exchange between the cytoplasmic pool and the pool at the flagellum base that would be sensitive to temperature or to flagellum length.

## Conclusion

In conclusion, although significant progress has been made in the identification of molecular actors of IFT and in the determination of their essential role for the construction of the flagellum, further studies are needed to understand several key steps. We believe that the combination of multiple models such as *Chlamydomonas* and *Trypanosoma*, but also of other species (*C. elegans*, *Drosophila*, *Tetrahymena*, and so on) with various biological and practical specificities will provide exciting answers to the questions raised in this review. For example, the fact that *T. brucei* assembles flagella varying from to 2 to 30 μm according to its stage of development [102] provides an opportunity to investigate the relationship between IFT and flagellum length. In contrast to most studies published so far where flagellum length was artificially modified, here it is the organism itself that alters the length of the organelle. Correlating the formation of these different flagella with the IFT activity (frequency, rate, and distribution) should illuminate the role of IFT in the control of flagellum length. Dissecting the multiple steps of IFT and the role of its individual components goes beyond basic research since it could provide key information to understand the significance of recently reported missense mutations affecting *IFT* genes in human patients suffering from ciliopathies.

## Abbreviations

DIC: Differential interference contrast; FC: Flagella connector; FRAP: Fluorescence recovery after photobleaching; GFP: Green fluorescent protein; IFA: Immunofluorescence assay; IFT: Intraflagellar transport; KAP: Kinesin-associated protein; ODA: Outer dynein arm; PFR: Paraflagellar rod; RNAi: RNA interference; RNA-seq: RNA sequencing; TEM: Transmission electron microscopy.

## Competing interests

The authors declare that they have no competing interests.

## Authors’ contributions

BM and PB conceived and wrote the paper. Both authors read and approved the final manuscript.

## Supplementary Material

Additional file 1: Table S1 Selected over-represented gene ontology terms found associated with transcripts that were cell-cycle regulated involved in the flagellum structure. Click here for file
